# Nephrogenic adenoma of the renal pelvis: A rare case report and review of the literature

**DOI:** 10.1097/MD.0000000000027025

**Published:** 2021-08-27

**Authors:** Feilong Zhang, Jiyue Wu, Zejia Sun, Dawei Xie, Xiaoyong Yang, Wei Wang

**Affiliations:** Department of Urology, Beijing Chaoyang Hospital, Capital Medical University, Beijing, China.

**Keywords:** immunohistochemistry, nephrogenic adenoma, rare case report, renal pelvis

## Abstract

**Rationale:**

Nephrogenic adenoma (NA) is a rare benign lesion of the urinary tract, which rarely occurs in the renal pelvis. Only 19 cases have been reported in the literature. However, there is no detailed report on the clinicopathological features of NA of the renal pelvis.

**Patient concerns:**

This case report describes a 46-year-old male patient who was admitted to the hospital for one month because of painless gross hematuria with blood clots. He had a history of hyperuricemia and a family history of gastric cancer.

**Diagnoses:**

NA of the renal pelvis was diagnosed pathologically and immunohistochemical.

**Interventions:**

The patient underwent laparoscopic nephroureterectomy.

**Outcomes:**

The patient recovered well after the operation with no discomfort. In addition, we followed up with the patient regularly post-discharge (approximately 20 months). There were no obvious abnormalities in the results of routine urine culture, computed tomography scan of the abdomen, and cystoscopy during the follow-up period, and the symptoms disappeared completely and did not recur.

**Lessons:**

NA of the renal pelvis is extremely rare in the clinic, which can be easily misdiagnosed and overtreated. However, for pathological diagnosis of this disease, specific immunohistochemical staining for preoperative biopsy was reported to be significant, which should be considered by the urologists and pathologists.

## 1 Introduction

Nephrogenic adenoma (NA) is a rare benign lesion, which can occurr in any part of the urinary tract from the renal pelvis to the urethra, mostly in the bladder, followed by the urethra and ureter, and rarely in the renal pelvis and prostate.^[1]^ NA is common in male adults and is related to chronic urinary inflammation, stones, and previous urological surgery.^[2]^ NA mimics a variety of urinary malignancies, and its clinical manifestations and urinary endoscopy are not specific. The differential diagnosis of NA requires a comprehensive analysis of histopathology and immunohistochemistry results. At present, the treatment of NA mainly involves minimally invasive endoscopic therapy. Additionally, there are cases of successful treatment of bladder NA in children and adults with sodium hyaluronate alone.^[3,4]^ However, after surgical removal of the lesion, NA can easily relapse; the recurrence rate of NA in children is the highest.^[5]^ In addition, NA tends to perinephric adipose tissue infiltration and malignant transformation, which needs to be considered by the clinicians.^[6,7]^ Thus, regular follow-up examinations post-operation are essential for patients with NA. Herein, we report for the first time a 46-year-old male patient presenting with painless gross hematuria, with no predisposing factors of renal pelvis NA, whose diagnosis was confirmed by histopathology and immunohistochemistry. Additionally, we also reviewed the literature related to NA and summarized all reports of renal pelvis NA, which would be helpful to improve and deepen our understanding of NA.

## 2 Case presentation

**Figure 1:**
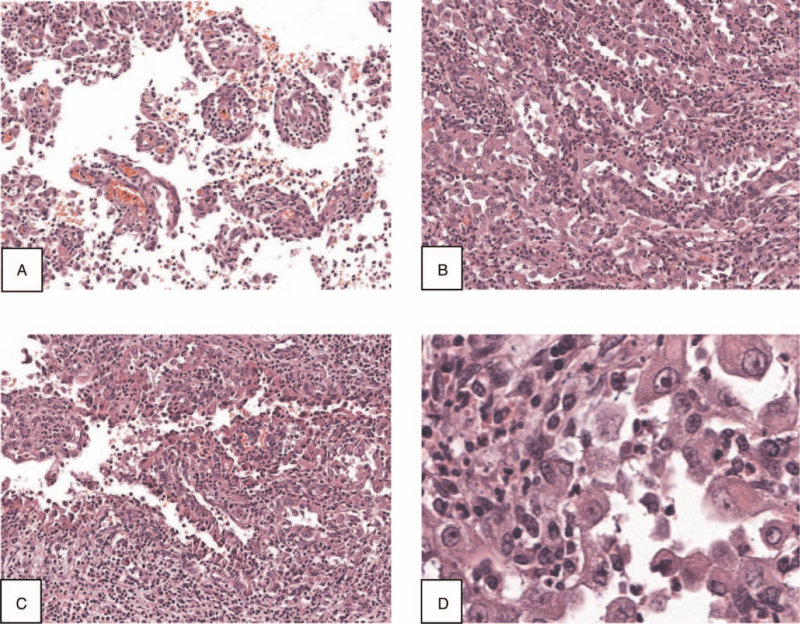
H&E stain of the nephrogenic adenoma of the renal pelvis. The lesion shows a mixed morphology with papillary (A) and tubular (B) pattern. Tumor cells are mainly monolayer cuboidal epithelium arranged around blood vessels, with hobnail-like cells (C) and enlarged epithelial nuclei, prominent nucleoli, and nuclear deviation (D) (magnification: A 10×; B 10×; C 10×; D 20×).

**Figure 2:**
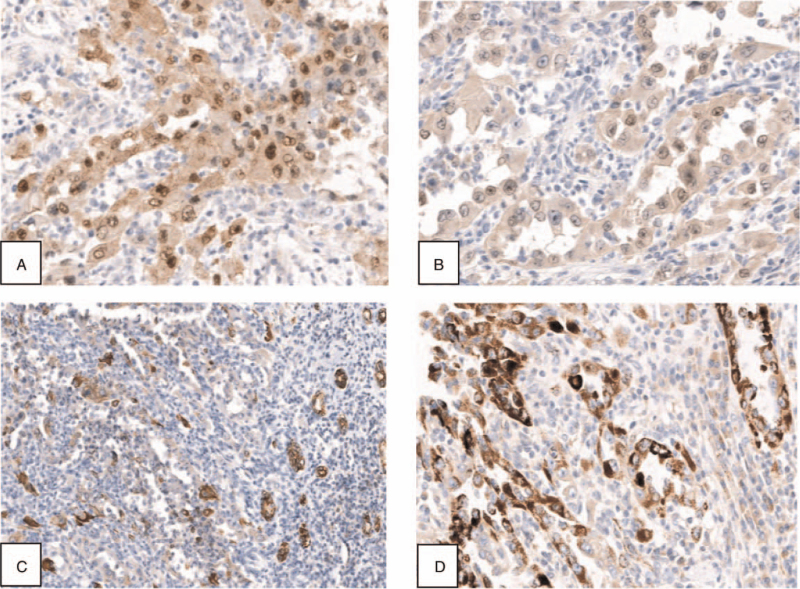
Immunohistochemical stain of the nephrogenic adenoma of the renal pelvis. (A) Positive staining with PAX8, (B) weakly positive staining with PAX2, (C) focally positive staining with CK7, and (D) positive staining for AMACR/P504S (magnification: A 20×; B 20×; C 10×; D 20×). AMACR = α-methylacylcoenzyme A racemase, CK = cytokeratin, PAX = paired box gene.

A 46-year-old male was hospitalized in the department of urology of our hospital, on October 24, 2019, due to intermittent painless gross hematuria with irregular blood clots that had persisted for 1 month. The patient showed no signs of fever, frequent and urgent urination, waist and abdominal pain, or dysuria. The medical history included hyperuricemia. In addition, the patient had a family history of gastric cancer. Cystoscopy showed bleeding from the left ureteral orifice and no obvious abnormalities in the bladder. Plain and enhanced pelvic computed tomography scans showed an isodense soft tissue mass of about 3.6 × 2.2 cm in the middle calyx and pelvis of the left kidney, with mild enhancement in the arterial phase, due to which urothelial carcinoma was considered. The results of search for tumor cells in urine thrice showed degenerative nuclear atypical epithelial cells. Laparoscopic nephroureterectomy was performed on October 31, 2019, under general anesthesia. The postoperative gross specimen showed a hard gray-yellow nodule protruding from the surface of the renal pelvis in a papillary shape, approximately 1.5 × 1.5 × 1.0 cm in size. Histopathological examination showed that the lesion exhibited a mixed papillary and tubular pattern; the tumor cells were mainly monolayer cuboidal epithelium arranged around blood vessels, with hobnail-like cells and enlarged epithelial nuclei, prominent nucleoli, nuclear deviation, with significant inflammatory cell infiltration around them (Fig. 1). The immunohistochemical assay showed positive staining for α-methylacylcoenzyme A racemase (AMACR)/P504S and renal tubular marker Paired box gene (PAX)8, weakly positive staining for PAX2, focally positive staining for cytokeratin (CK)7 (Fig. 2), but negative staining for P63 and GATA3. Combined with the results of histopathological and immunohistochemical staining, the final diagnosis was reported as NA of the renal pelvis. The patient was followed up regularly after discharge, around 20 months. There were no obvious abnormalities in the results of routine urine culture, computed tomography scan of the abdomen, and cystoscopy during the follow-up period; the symptoms disappeared completely and reported of no discomfort before publishing this article.

## 3 Discussion and conclusions

NA was originally described as a benign hamartomatous lesion in the bladder.^[8]^ The term “nephrogenic adenoma” was proposed in 1950 by Friedman and Kuhlenbeck because these lesions were histologically similar to renal tubules.^[9]^ NA typically occurs in male adults, with the male to female ratio of approximately 2:1.^[10]^ There are some risk factors for NA, including genitourinary trauma, mechanical stimulation or genitourinary surgery, local chemical stimulation, radiation, chronic inflammation, immunosuppression, and renal transplantation.^[2,11]^ In addition, NA is common in patients with augmented bladder.^[12]^

At present, there are 3 theories about the origin of NA. According to the “inflammatory theory”, chronic inflammation and stimulation of urothelial cells can lead to the metaplasia of urothelial cells to nephrogenic cells, which is characterized by NA. The “embryological theory” holds that NA is caused by embryogenic mesonephric tissue remaining in the bladder. However, the “implantation theory” believes that the occurrence of NA is caused by the seeding of exfoliated renal tubular cells on the damaged mucosal surface. Mazal et al^[11]^ used fluorescent in situ hybridization to confirm that the bladder NA of the renal transplant recipient originated from the renal tubular cells of the transplanted kidney not from the bladder urothelial mucosa of the recipient. At least in the case of renal transplantation, the occurrence of NA is known to be related to the implantation of renal tubular cells in the bladder; however, how implantation of renal tubular cells occurs after seeding and the specific mechanism needs further clarification.

NA can imitate various malignant urinary tumors, especially urothelial cancer, which can result in misdiagnosis. The clinical and endoscopic manifestations of NA are not specific, and the clinical manifestations of NA in different locations are different. The manifestation of NA in the urethra and prostate may present as dysuria symptoms; bladder NA mainly manifests as irritation symptoms, such as frequent and urgent urination; ureteral and renal pelvis NA is usually asymptomatic and may present as hematuria, often accompanied by predisposing factors of NA, the most common one being calculi. Here, we reported for the first time that a patient presenting painless gross hematuria with no predisposing factors was diagnosed NA of the renal pelvis, which should be noted by urologists and pathologists.

Histological examination can be helpful for the diagnosis of NA; it is essential to understand the characteristic morphological manifestations of NA. The histological morphology of NA is diverse, including traditional types of tubular, tubulocystic, polypoid, and papillary patterns. Hansel et al^[13]^ first described a fibromyxoid variant of NA in 2007, after which the first case of fibromyxoid NA in the ureter was reported.^[14]^ In addition, a flat pattern was first described in 2013, and was found in 8 of 15 cases of NA, often mixed with the more traditional tubular, polypoid, and papillary components.^[15]^ Besides, they also found that flat pattern was a common histomorphological manifestation of NA but could be easily confused with atypical flat urothelial lesions.^[15,16]^ In this case, the histomorphology showed a mixed arrangement of papillary and tubular patterns. Due to the diversity of NA morphology, this benign lesion could mimic papillary urothelial carcinoma, metastatic tumor, and clear cell adenocarcinoma.^[17]^ Thus, immunohistochemical staining and characteristic morphological features were found to be helpful for the identification of NA.

Immunohistochemical staining is of great significance for the differential diagnosis of NA. The typical immunohistochemical expression of NA was positive for CK7, a cluster of differentiation 10, AMACR/P504S, epithelial membrane antigen (EMA), cannabinoid 1, PAX2, and PAX8, but negative for p63, prostate-specific antigen, and carcinoma embryonic antigen.^[2,18]^ PAX2, a key renal transcription factor, is known to be widely expressed in NA and has diagnostic specificity. Therefore, some researchers believed that this study supported the origin of NA from renal tubular cells. PAX2 and PAX8 are known to be related to the development and proliferation of renal tubules, and several studies have shown that PAX2 and PAX8 are almost 100% expressed in NA.^[15,19]^ It has also been reported that PAX8 is the most reliable marker for the diagnosis of NA^[18]^ but early work by Pellizarri et al^[20]^ found that 93% of non-invasive bladder urothelial carcinomas expressed PAX8. Additionally, some studies reported that PAX8 was also expressed in some bladder urothelial carcinomas.^[21,22]^ A new study found that Napsin A was a highly sensitive marker of NA, and it could be used as a useful auxiliary marker of PAX8 when diagnosing NA.^[23]^ For the differential diagnosis of NA, the combined detection of PAX8^+^, p63^-^, and EMA^+^ could distinguish NA from the most common urothelial and prostate carcinoma.^[24]^ In addition, Napsin A combined with PAX8 was found to have a high specificity for the differential diagnosis of NA from simple urothelial carcinoma and prostate cancer.^[23]^ It was confirmed that the combined detection of immune markers AMACR, CK903, and P63 could reduce the possibility of NA being misdiagnosed as prostatic adenocarcinoma.^[25]^ GATA3 is a sensitive and specific marker for urothelial carcinoma and can distinguish NA from other tumors in the differential diagnosis. However, about 40% of NA reported in the literature are positive for GATA3.^[25]^ The immunohistochemical results of this case showed positive staining for PAX2, PAX8, AMACR/P504S, and CK7, while negative staining for P63 and GATA3, which was consistent with the immunohistochemical characteristics of NA. Therefore, GATA3 should be used cautiously when differentiating NA from urothelial carcinoma. At present, the differential diagnosis of NA requires a comprehensive judgment of immunohistochemical results combined with multiple markers.

Currently, the main treatment of NA is minimally invasive resection of local lesions, which not only helps to confirm the diagnosis but significantly relieves the symptoms of patients. Among them, NA occurring in the prostate is transurethral resection of the prostate; NA occurring in the urethra and bladder is mainly transurethral mass resection; ureteroscopic holmium laser resection of adenoma is the main therapeutic method of ureteral NA; flexible ureteroscopic holmium laser resection and tumor resection under nephroscope are feasible for NA of the renal pelvis. In addition, the latest study has reported a case of obstructing ureteral NA in a child. Due to the ureteral location and extensive NA disease involvement, Taylor C. Peak et al^[26]^ performed an ileal ureter interposition with ureterectomy, believing that this was a safe and effective option and could reduce the risk of recurrence in the future. In addition to surgical treatment for NA, Campobasso et al^[3]^ successfully treated a 12-year-old boy diagnosed with bladder NA with sodium hyaluronate. Then several reports subsequently confirmed that NA could be successfully treated by injecting sodium hyaluronate into the bladder of children with bladder NA.^[27]^ A recent study also reported the first case of injection of sodium hyaluronate in the bladder to successfully treat adult NA,^[4]^ indicating that sodium hyaluronate could be an alternative treatment for patients who were not suitable for surgical resection of lesions. Currently, more case reports are needed to provide further evidence that topical application of sodium hyaluronate as an effective treatment option.

NA is prone to recurrence; the recurrence rate of NA in children is the highest, approximately 80%,^[5]^ which might be related to incomplete resection of a flat pattern of NA or re-implantation of new renal tubular cells after surgery. The correct identification of the susceptibility factors of NA and then intervention might be helpful for the treatment of NA and may reduce its recurrence, since the literature suggests that treating cystitis alone might be sufficient to resolve bladder NA.^[4]^ Bladder NA is usually confined to the lamina propria, but sometimes it can locally involve the superficial muscularis propria.^[28]^ When NA involves the renal pelvis and ureter, it almost always only involves the lamina propria. However, there are also reports of NA infiltrating deep into the perinephric adipose tissue, which should be paid more attention by pathologists.^[6]^ NA hardly develops into malignant progression and metastasis, but it has been reported that bladder NA could transform into moderately differentiated bladder adenocarcinoma after repeated resection of the lesion.^[29]^ Hartmann et al^[7]^ also reported that a 70-year-old female patient with bladder NA subsequently progressed to clear cell adenocarcinoma after multiple recurrences. Therefore, clinicians should be aware that this rare benign tumor can undergo malignant transformation. Long-term postoperative follow-up and regular cystoscopy are necessary to identify early signs of NA recurrence and malignant transformation.

In conclusion, NA of the renal pelvis is relatively rare, and its clinical manifestations and endoscopy are very similar to those of renal pelvis cancer, which can be easily misdiagnosed and require more attention from clinicians and pathologists. At present, 19 cases of renal pelvis NA have been referred in the literature, but they do not provide detailed reports of renal pelvis NA.^[6,16,24,30–33]^ We present the first study to systematically introduce and summarize renal pelvis NA (Table 1). According to this case report, urologists and pathologists should consider the possibility of NA of renal pelvis in the diagnosis of renal pelvis tumors with painless gross hematuria. Characteristic histomorphology and specific immunohistochemical examination can be helpful for the differential diagnosis. Due to the rarity of NA, there are no randomized controlled trials on NA so far. Therefore, there is no unified standard for the treatment of NA, and it is necessary to conduct multi-center prospective trials to determine the optimal treatment of NA and provide a reference for reducing its recurrence.

**Table 1:** Summary of the reported cases of nephrogenic adenoma of the renal pelvis.

Year	Country	Cases	Age	Sex	Predisposing factors	Treatment	Follow-up	Recurrence	Reference
1991	Spain	1	36	Male	Renal calculi, repeated genitourinary surgery	Endopyelotomy	-	-	^[30]^
1992	Spain	1	-	-	-	-	-	-	^[31]^
1993	Germany	1	35	Female	None	Partial nephrectomy	-	-	^[32]^
2013	America	2	73	Female	Genitourinary surgery	Nephrectomy	5 months	None	^[6]^
			20	Female	Renal calculi	Pyelolithotomy	1 month	None	
2013	Spain, Italy, the Czech Republic	11	-	-	-	-	-	-	^[24]^
2013	China	1	48	Female	None	Tumor resection under nephroscope	12 months	None	^[33]^
2017	Turkey	2	-	-	-	-	-	-	^[16]^

## Author contributions

**Conceptualization:** Xiaoyong Yang.

**Formal analysis:** Jiyue Wu.

**Supervision:** Zejia Sun, Dawei Xie, Xiaoyong Yang.

**Writing – original draft:** Feilong Zhang.

**Writing – review & editing:** Jiyue Wu, Wei Wang.
